# Comparative analysis of ageing in Nigeria and United Kingdom using life course approach: the implication for the Nursing profession in Nigeria

**DOI:** 10.11604/pamj.2021.38.411.22272

**Published:** 2021-04-29

**Authors:** Nafisat Oladayo Akintayo-Usman, Saheed Opeyemi Usman

**Affiliations:** 1 Faculty of Health Sciences and Sport, University of Stirling, Stirling, United Kingdom,; 2 Department of Chemical Pathology, Nnamdi Azikiwe University, Awka, Nigeria

**Keywords:** Gerontology, nursing, healthy ageing

## Abstract

The population of Nigerian older persons is expected to reach 26 million by 2050 from 9 million reported in 2016. This population change has several implications, thus the need to pay attention to healthy ageing. Hence, this report aims to compare ageing and its facilitators in Nigeria and the United Kingdom (UK). The life course theory was used to explore the influence of early life factors and experiences on ageing. Unlike the UK, little attention is given to the care of Nigerian older persons. Therefore, Nigerian stakeholders must design and implement a comprehensive policy on healthy ageing. Also, there is an urgent need for training nurses to meet this demand as it arises.

## Essay

### Introduction

The population is ageing globally, with an estimated 962 million older persons in the world and a growing rate of about 3% yearly [1,2]. By 2050, the world´s population of older persons is expected to reach 2 billion [1]. Likewise in sub-Saharan Africa, the number of older persons is expected to increase over threefold, from 46 million in 2015 to 147 million in 2050 [3]. In Nigeria, there were 2.1 million older persons in 1999; compared to 9 million reported in 2016 [4,5]. This change in population structure has several implications on their well-being [6]; thus, the need to pay attention to healthy ageing.

Ageing is a complex multifactorial process characterized by progressive changes in body tissue, which eventually lead to a decline in function and death of the individual [7]. There are several approaches to explain ageing. One of the most prominent theoretical approaches is the life course approach, with the view that ageing takes place across the entire lifespan [8]. The authors compared ageing in two countries, one developed and developing country each, using life course theory as a framework. Also, factors affecting healthy ageing in the two countries were explored. Nigeria was chosen as a case study; comparing it to United Kingdom (which colonised Nigeria). The two countries presently have the largest population of older persons in Western Africa and Northern Europe respectively [9].

### Ageing in the two countries

The life course theory was chosen to compare ageing in the two countries, because of the role of location in its framework [10]. The population of older persons in Nigeria and the UK are both increasing - though at a different rate. Nigerian older population is expected to grow from over 9 million in 2016 to 26 million by 2050 [5,9]; while the UK older population is expected to increase from 15 million in 2017 reach 23 million by 2050 [9]. There is therefore a need to pay attention to healthy ageing. According to the theory, healthy ageing starts from preconception and takes place across the entire lifespan.

Accelerated ageing has been traced to early life [11]. The neonatal mortality rate in Nigeria is 33 deaths per 1000 live births, compared to 3 deaths per 1000 live births in the UK; while the under-5 mortality rate per 1000 live birth was 100.2 in Nigeria compared to 4.3 in the UK [12]. This shows a wide gap in children´s wellbeing in these two countries, which may not be unconnected to the quality of care received by their mothers during antepartum, delivery and puerperium. Though every woman likes to experience a positive birth experience, only 37% of Nigerian women with a live birth delivered in a health facility [12,13]. These women even complained of been mistreated during childbirth in the health facilities [14]. A similar report revealed 40% of births were attended by skilled health personnel in Nigeria, whereas 99% of births in the UK were attended by skilled health personnel [12]. Such negative experiences by Nigerian women can hurt the health of the neonates.

Malnutrition is also a major health challenge in childhood. It was reported that 50% of Nigerian under-five children are malnourished, with 13.1 million children stunted and 2.9 million wasted; while 30% of Nigerian children aged 6 to 23 months are living on poor diets [15]. Though better than Nigeria, UK is also facing a rising tide of malnutrition. It was reported that 10% of UK children are living with severe food insecurity [16]. Besides, UK was ranked the second country with the highest rate of obesity in Europe, putting such children at risk of non-communicable diseases as they grow older [16]. In addition to the early life adversity, early reproduction has also been linked to accelerated ageing [11]. Child marriage is a problem in Nigeria. It has been reported that 18% of Nigerian girls are married by the age of 15 years; 44% by the age of 18 years; 3% of Nigerian boys by the age of 18 years [17]. Though there is data scarcity on the prevalence of child marriage in the UK, the age of consent in the UK is 16 years, while it is 11 years in Nigeria - the lowest age of consent globally [18]. This is one of the major factors responsible for the high prevalence of child marriage in Nigeria [18]. Child marriage thus exposes these teens to teenage pregnancy and motherhood.

Meanwhile, teenage pregnancy and motherhood have both health and social consequence. Children born to such mothers are at increased risk of sickness and death, while the mothers are more likely to experience adverse pregnancy outcomes and difficulty in pursuing educational opportunities [19]. UK´s teenage pregnancy rate is 17.9 conceptions per 1000 women, 51.7% of which resulted in abortion; while the fertility rate among Nigerian teenagers has been reported to be 107 births per 1000 women [19,20]. Yet, only 40% of births were attended by skilled health personnel in Nigeria, while 99% in the UK [12]. This makes Nigerian teen mothers more exposed to hazards during labour and delivery than their counterparts in the UK. Teenage pregnancy and motherhood also abrupt their education with consequent exposure to poverty. Both illiteracy and poverty are inimical to healthy ageing. Literacy is linked to healthy ageing through a range of socio-economic factors and health. Over 14 thousand and 11 million children are out of school in the UK and Nigeria respectively - ranking Nigeria first among 126 countries compared [21]. Likewise, 51.4% of Nigerians and less than 3% of the UK population live in multidimensional poverty [22,23]. Such a poverty level should not be unexpected in Nigeria with a 23.1% unemployment rate, compared to 3.8% in the UK [24].

Moreover, an unhealthy lifestyle predisposes an individual to non-communicable diseases (NCDs) and ageing [25]. Both countries now experience unhealthy lifestyle among their adults. Among adults, the obesity rate is 8% and 30% in Nigeria and UK respectively; tobacco smoking rate is 6% and 11% in Nigeria and UK respectively; physical inactivity rate is 25% and 38% in Nigeria and UK respectively [26]. It was further reported 29% and 89% of all deaths in Nigeria and the UK respectively are as a result of NCD, especially diabetes, chronic respiratory diseases, cancers and cardiovascular diseases [26]. This report explains why unlike in the past when NCDs were a burden in developed countries like the UK, it is now a burden to developing countries like Nigeria too [25].

### Facilitators of healthy ageing

The older persons´ most urgent concerns globally are healthcare and income security [27]. The two factors are germane to healthy ageing at old age. Availability of quality healthcare services facilitates healthy ageing. Meanwhile, older persons make significant use of health care services - because of the higher rates of morbidity and disability occurring at old age [28]. Older persons must have access to age-friendly and affordable healthcare information and services that meet their needs [27]. From the life course perspective, health care services should include health promotion and disease prevention activities [27]. National Health Insurance Scheme, NHIS, is the agency saddled with the responsibility of ensuring every Nigerian has easy access to healthcare. There are many programmes within the NHIS, including the Vulnerable Group Social Health Insurance Programme (VGSHIP) - which is designed to provide healthcare to the vulnerable including the older persons [29]. However, over 90% of Nigerians are living with no health insurance coverage [30]. This is because the NHIS focuses more on the formal sector workers; meanwhile, up to 90% of the working population are in the informal sector [31]. Hence, the majority of the population access healthcare out of pocket. Similarly in the UK is the National Health Service, NHS, which ensures all residents have access to healthcare regardless of their socioeconomic status. Unlike NHIS, coverage is universal [32]. Nonetheless, 7% of UK people could not access care because of healthcare cost [33]. Be that as it may, UK performed far better in the NHS programme, compared to Nigeria´s NHIS.

Some of the major contributory factors to difficult access to healthcare among older persons are low socioeconomic status, low health literacy and ethnic minority in the UK; while high poverty level and poor road network in Nigeria [22,34,35]. Another main factor is the availability of health workers. The number of doctors per 1000 people is 0.4 and 2.8 in Nigeria and UK respectively; while that of nurses and midwives is 1.5 and 8.3 respectively [36]. In addition to the availability of health workforce, is their training. Training health care professionals is pertinent to ensure caregivers have access to information and basic training in the care of older people [27]. Unlike Nigeria, UK has many gerontological and geriatric programmes for its health workforce.

As important as healthcare is to healthy ageing, so also is economic security. There is a threat to the sustainability of healthy ageing because people experience earlier retirement and enhanced longevity [28]. In addition to the threat, is the global economic crisis - which has exacerbated the financial pressure to ensure economic security in old age [27]. Hence, the concern of economic security at old age. Older persons are more likely to be income poorer than the rest of the population [37]. With the high poverty level in Nigeria, retirees´ vulnerability to economic hardship is high [38]. Hence, the pension system is one of the most important ways to ensure economic independence and reduce poverty in old age - thereby, facilitating healthy ageing [27]. In developing countries, like Nigeria, social insurance and pension systems mostly cover formal employment only. This makes coverage a great challenge, as a larger proportion of the workforce is in the informal sector [27]. More so that there is no national security system for older persons in Nigeria [38]. Unlike the developing countries, social insurance and pension system are working in developed countries like the UK, though sustainability is a major concern [27]. Aside from the pension system, many countries are increasing their retirement age to improve the financial sustainability and pension adequacy of older persons [39]. Older persons are very productive; they can work efficiently and contribute to national development if the right measures are in place [27]. This raises the issue of flexible retirement - the ability to draw a pension while continuing in paid work, often with reduced working hours, or to choose when to retire [39]. While post-retirement engagement is on the rise for older persons in the UK, it is difficult for the Nigerian government to encourage flexible retirement with the 17.6 million unemployed Nigerian youth [30,39]. Thus, active ageing is easier in the UK than in Nigeria.

### Implication for nursing profession in Nigeria

By 2050, with 26 million Nigerians and 23 million UK citizens reaching old age, Nigeria will have more older persons than the UK [9]; yet, we are less prepared for the care of our older population. This is a great challenge for Nigeria, a country in which gerontological nursing is yet to emerge. Thus, there is an urgent need for training nurses to meet this demand as it arises because nurses are always in contact with older persons at all levels of care [40]. Many of these older persons live with multimorbidity and frailty. Yet, the family, often the primary caregiver and major source of social support is now giving its function to external agencies like geriatric homes. Today, there is no single gerontological nurse in almost all the geriatric homes in Nigeria. Moreover, the majority of our health facilities neither have a geriatric speciality unit nor a geriatric ward to offer specialized care to this population. The training of nurses in gerontology is therefore essential to the successful creation of geriatric specialities across our health facilities. Thus, the need for the Nursing and Midwifery Council of Nigeria to review the basic nursing school´s curriculum to include gerontological nursing. Similarly, more attention should be given to gerontology when organising continuing education programmes for licensed nurses. Lastly, there is a need for gerontological nursing as a speciality at the postgraduate level.

### Conclusion

With the rise in the Nigerian ageing population, there is an urgent need for training health workers who can meet the demand as it arises. Universal Health Coverage is key to healthy ageing. Luckily, nursing is the only profession that cares for people from preconception to death. Hence, the relevance of nurses in the life course approaches. There is a need for nurses to advocate for an overhaul of the Nigerian healthcare system, as the need for functional health care and health insurance systems is paramount to the wellbeing of Nigerians. Though the burden of NCDs is more enormous in the UK, there is a need to pay more attention to the menace of NCDs in Nigeria too. It is also imperative for all stakeholders to put all necessary measures in place, to elaborate the training of nurses on gerontology to improve the quality of care rendered to older persons.
